# Effects of Different Types of Exercise on Kidney Diseases

**DOI:** 10.3390/sports10030042

**Published:** 2022-03-10

**Authors:** Hamid Arazi, Majid Mohabbat, Payam Saidie, Akram Falahati, Katsuhiko Suzuki

**Affiliations:** 1 Department of Exercise Physiology, Faculty of Sport Sciences, University of Guilan, Rasht 4199843653, Iran; m67mohabbat@gmail.com (M.M.); saidie-p@guilan.ac.ir (P.S.); akram.falahati@gmail.com (A.F.); 2 Faculty of Sport Sciences, Waseda University, Tokorozawa 359-1192, Japan

**Keywords:** aerobic exercise, resistance exercise, electrical muscle stimulation, kidney disease

## Abstract

The effects of exercise on kidney function have been studied for more than three decades. One of the most common health issues among patients with chronic kidney disease (CKD) is a lack of physical activity, which leads to a low exercise capacity in these patients. The majority of maintenance hemodialysis (MHD) patients do not exercise at all. At each stage of dialysis, patients lose 10–12 g of their amino acids through blood sampling. Dialysis also leads to increased cortisol and circadian rhythm sleep disorders in hemodialysis (HD) patients. Studies have also reported higher C-reactive protein levels in HD patients, which causes arterial stiffness. Exercise has a variety of health benefits in these patients, including improved blood pressure control, better sleep, higher physical function, and reduced anxiety and depression. On the other hand, it should be noted that intense exercise has the potential to progress KD, especially when conducted in hot weather with dehydration. This review aimed to investigate the effects of different types of exercise on kidney disease and provide exercise guidelines. In conclusion, moderate-intensity and long-term exercise (for at least a 6-month period), with consideration of the principles of exercise (individualization, intensity, time, etc.), can be used as an adjunctive treatment strategy in patients undergoing dialysis or kidney transplantation.

## 1. Introduction

Chronic kidney disease (CKD) is a general term used for various disorders in the structure and function of the kidney. CKD is one of the leading causes of morbidity and mortality, with a global estimated prevalence of 13% (11–15%) [1]. A decline in the estimated glomerular filtration rate (eGFR) is a major indicator of kidney disease [2]. The normal range of GFR, adjusted for body surface area, is 125 mL/min/1.73 m^2^ in men [3]. In CKD, the eGFR is reduced to less than 60 mL/min per 1.73 m^2^. [4]. The most severe form of this disease is end-stage kidney disease (ESKD), during which the GFR drops to less than 15 mL/min per 1.73 m^2^, a stage at which dialysis or a kidney transplant is required to maintain the patient’s normal life [5,6]. CKD presents a variety of symptoms, including circadian rhythm sleep disorders, cardiovascular problems, a severe decrease in muscle mass, a decrease in antioxidant capacity, the disruption of the redox balance, and high blood pressure [2,6,7,8].

Lifestyle changes, the most important of which is exercise, are among the main strategies for the prevention and treatment of CKD, such as pharmacological approaches, dietary improvements, and antihypertensive drugs. Exercise has been recognized as a fundamental component for enhancing or maintaining overall health and wellness; improving physical function; and preventing and curing various diseases, namely reducing the risk of cardiovascular events and mortality [9] and possibly providing multiple benefits to CKD patients [2,4,10]. What is certain is that exercise affects the body’s various systems by altering the blood flow and metabolism, improving the body’s ability to utilize oxygen and increasing the demand for nutrients [7,9,10]. Kidneys and collecting ducts, muscles, and the cardiorespiratory system are among the body organs that change the way they work due to exercise [7,11,12]. Exercise and physical activity lead to changes in kidney hemodynamics and electrolytes [7,10,13]. Several studies have shown that exercise can improve eGFR and kidney function [4,6,13]. Toyama et al., investigated whether exercise therapy has the potential to modify lipid metabolism and improve the eGFR in patients with cardiovascular disease and CKD [14]. Furthermore, a meta-analysis confirmed that the eGFR was increased by 2.62 mL/min/1.73 m^2^ after exercise therapy in non-dialysis CKD patients [15]. A systematic review reported that aerobic and/or resistance training has significant positive effects on physical and functional capacity in non-dialysis CKD [16]. Hypertension, diabetes, and obesity are the risk factors that increase the incidence of CKD [15]. According to a meta-analysis, exercise therapy has anti-hypertensive effects, reducing both systolic and diastolic blood pressure (BP) in CKD patients [15]. In a study by Aoike et al., aerobic training improved BP in overweight non-dialysis-dependent CKD patients [17]. Contrarily, a lack of an impact of exercise on BP in CKD patients has been reported [14,18], which may be related to the type or intensity of exercise performed. During exercise, the effective renal blood flow (RBF) decreases, the degree of which is directly related to the intensity of exercise and sometimes reaches 25% of the resting value during strenuous exercise [6]. The potential health benefits of moderate-intensity exercise in reducing insulin resistance, metabolic syndrome, and chronic diseases is well-known [19]. Although moderate-intensity exercise is safe, high-intensity exercise may increase biomarkers indicative of muscle and kidney damage, namely serum creatine kinase (CK) and urine myoglobin and creatinine levels [19,20]. On the contrary, it was shown that HIIT is practical and safe for CKD patients, with similar benefits of HIIT and MICT on exercise capacity and skeletal muscle protein synthesis [21].

Now, considering the dual role of exercise in health, this study aims to investigate the feasibility and potential benefits of different types of exercise in CKD patients. The investigation of the effects of various types of exercise on physical function and different health aspects in patients with different kidney diseases is clinically important because there are distinctions between kidney diseases.

## 2. Materials and Methods

**Figure 1:**
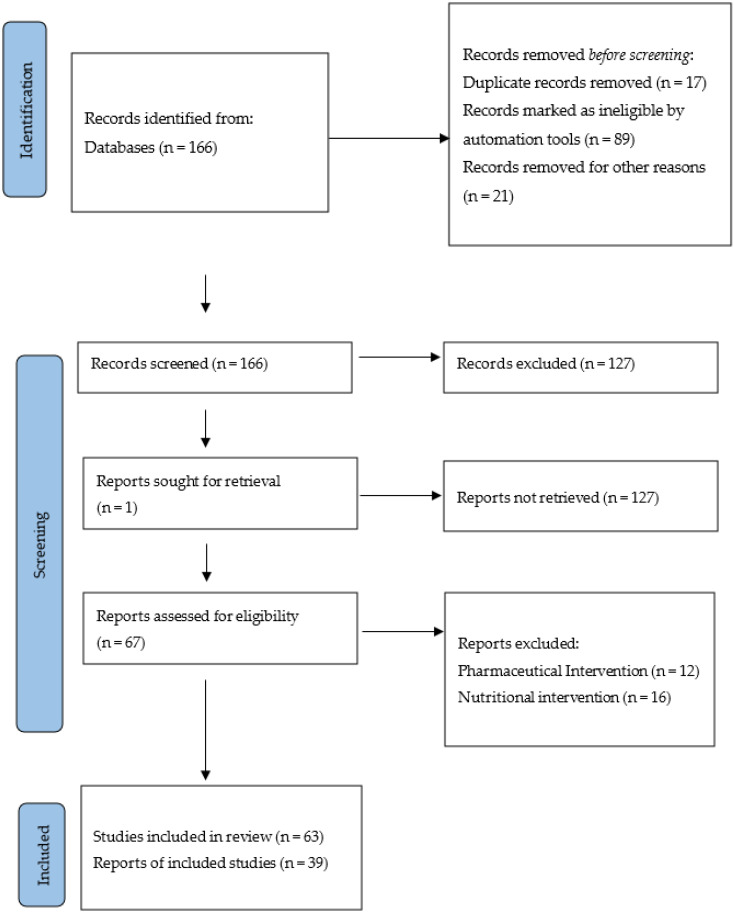
Review methodology scheme for the search results.

PubMed and Google Scholar were electronically searched for papers that were published in English. The search strategy included different combinations of the terms “CKD” and “ESRD” with exercise intervention terms such as “exercise”, “aerobic exercise”, “resistance exercise”, “combined exercise”, “exercise with blood flow restriction”, “physical activity”, “functional exercise”, “electrical muscle stimulation”, or “high-intensity interval training (HIIT)”. Inclusion criteria were clinical trial studies and randomized and controlled clinical trials. Only full-text articles were selected. The searching procedure was performed by two independent authors (M.M. and A.F.). Upon the occurrence of any possible disagreements as to whether a particular study should be considered in the study, the opinion of the supervising professor was asked. The next step was to read the full text of the considered studies. A total of 166 articles were found with the above criteria, and after screening, 39 of them were examined: a total of 18 for aerobic exercise interventions, 7 for resistance exercise, 5 for combined exercise, 3 for exercise with blood flow restriction, 3 for electrical muscle stimulation, 2 for HIIT, and 1 for functional exercise. Exclusion criteria included studies that used a combination of nutritional and exercise interventions and drug and exercise interventions, and estimation methods. A total of 1449 patients with CKD in the early stages of ESRD and patients with kidney transplantation were included in this study. Most of the applied interventions were intradialytic, and some were home-based exercises. Moreover, resistance exercises in these studies were performed using elastic bands. We excluded animal studies; furthermore, most of the human studies were carried out on adults. See Figure 1.

## 3. Results

### 3.1. Aerobic and Functional Exercise in Kidney Diseases

Aerobic training such as cycling, walking, and swimming is the most popular form of exercise training. Cardiovascular disease (CVD), namely coronary artery disease, heart failure, arrhythmias, and sudden cardiac death, are highly prevalent in CKD [22]. A systemic, chronic pro-inflammatory state resulting in vascular and myocardial remodeling, processes causing atherosclerotic lesions, vascular calcification, and vascular senescence, as well as myocardial fibrosis and the calcification of cardiac valves, are common complications in CKD [22]. The prevalence of these diseases is remarkably higher in patients with advanced CKD (stages 4–5) compared with early CKD (stages 1–3) and the general population.

The effects of aerobic exercise on the blood pressure (BP) and cardiovascular system of CKD patients are conflicting. Jeong et al., investigated the effect of aerobic exercise (30 min cycling) during a hemodialysis session. The exercise intensity was equal to 11–13 of the 6–20 Borg rate of perceived exertion (RPE). Stroke volume, cardiac output, and heart rate increased transiently during exercise, but aortic and brachial artery blood pressure, as well as autonomic nerves, were the same as non-exercise days [8]. In another study, Cooke et al., examined the effect of intradialytic pedaling exercise on arterial stiffness in hemodialysis patients. The study consisted in a 4-month training program with three sessions per week until an intensity equal to 12–16 RPE (mean 42.6 min) was reached. In this study, the carotid–femoral pulse wave velocity (CfPWV) was used as a reference for atherosclerosis. The results showed a larger absolute decrease in CfPWV. Heart rate also decreased after the training intervention. The augmentation index (AIx), which is an indicator of wave reflection at the time of pulse measurement, increased in the non-training group and remained unchanged in the training group. CfPWV returned to the previous state after the training period [23]. In the study by Belik et al., aerobic exercise training (4-month; three sessions per week for 30–45 min each with an intensity equal to 65–75% HRmax) improved cerebral blood flow [10]. In another study, Kirkman et al., examined the effect of aerobic exercise on vascular function in non-dialysis patients with CKD. The exercise intervention included 12 weeks of aerobic training (cycling, walking, jogging, or elliptical), three times per week with an intensity equal to 60–85% of the heart rate reserve (HRR), which progressively increased to 45 min. A heart rate monitor and RPE of 12–16 were used to set the intensity. The control group received the routine standard care. The results showed that 12 weeks of moderate to vigorous aerobic training enhanced cardiorespiratory fitness (assessed by VO_2_peak and cardiopulmonary exercise testing), preserved conduit artery endothelial function (evaluated by brachial artery baseline vs. peak diameter and shear stress area), and advanced microvascular function (determined by skin blood flow response to local heating over time) in patients with CKD. Nonetheless, arterial stiffness (mean arterial blood pressure, central systolic and pulse pressure, the augmentation index, forward or reflected pulse wave amplitudes, or arterial pulse wave velocity) did not change post-training [12]. In another study, Silva et al., examined the effect of aerobic exercise on non-traditional cardiovascular risk factors in hemodialysis patients. The exercise was performed three times a week for 4 months, with an intensity equal to 65–75% of the HR or near 13 RPE. The results showed that the diameter of the septum decreased in the exercise group and potassium was increased in both groups. These findings also indicated that exercise maintained the status of C-reactive protein, because this factor was higher in the control group [24]. This result was consistent with the findings of Graham-Brown et al., who reported left-ventricular volume reduction after 6 months of cycling for 30 min per day, three times a week [25]. In a systematic and meta-analytic study, Huang et al., examined exercise in hemodialysis patients. Interventions included aerobic exercise and resistance training or combined training, which ranged from 8 weeks to 12 months. The frequency of training (days per week) was often three sessions. Exercise intensity was moderate and peaked between 55–60% of the peak power. The duration of exercise in each session (training volume) varied from 15 to 90 min. Blood pressure was assessed in 7 studies, which included 137 patients in the experimental group and 123 patients in the control group. The result of the study, inconsistent with other results, showed that resistance training may not be useful for dialysis. The results also displayed that diastolic blood pressure did not decrease due to aerobic and combined exercise [26]. Data are summarized in Table 1.

In another study, aerobic capacity was analyzed using peak oxygen uptake (VO_2max_), defined as the maximum amount of oxygen that the body can absorb, transport, and consume per unit time. VO_2max_ was assessed on a treadmill during the initial test. The training intensity was 50–60% of VO_2max_. The results showed that VO_2max_ improved by 20% [28]. Since dialysis increases CO_2_ production and excretion in hemodialysis patients and leads to a decrease in pulmonary leukocytes, aerobic exercise, especially when acute, can prevent increasing hydrogen ions with alkalosis, which leads to increased oxygen consumption. On the other hand, aerobic exercise leads to an increase in hemoglobin, oxygen saturation, and oxygen parabolic pressure, and due to its contribution to angiogenesis and increase in central blood flow, improves capillary exchanges, especially in the lower limbs, which are immobile during dialysis [7]. Moreover, the pulmonary circulating leukocytes in these patients accumulate in pulmonary vessels due to blood contact with the dialyzer membrane, leading to microatelectasis and reducing cardiac output. Therefore, the increase in cardiac output induced by aerobic exercise can lead to an improved condition [7]. In another study, Pomidori et al., examined the effect of a minimal dose of exercise (two 10 min walking sessions every other day at an intensity less than the self-selected speed) compared with the control group (usual care) on respiratory muscle strength and lung function. The exercise protocol improved the physical fitness (6 MWT) and reduced the ventilatory work of hemodialysis patients [29].

One of the most crucial pathophysiological mechanisms for CVD in CKD patients is the facilitated formation of atherosclerotic plaques due to hyperlipidemia, uremic toxins, inflammation, oxidative stress, and endothelial dysfunction [30]. In CKD, a high serum phosphate concentration is connected with a deterioration in renal function. In early CKD, because of the increased fractional excretion of phosphate, the serum phosphate concentration is normal. Phosphaturia results in renal tubular damage associated with inflammation and oxidative stress [31]. In a study by Manfredini et al., patients walked for 20 min three times a week with low to moderate intensity. Biochemical factors related to kidney function such as creatinine, phosphate, albumin, and calcium, and lipid profiles such as cholesterol, triglycerides, and glucose did not change. In this study, the time to perform 5 STS test was decreased, and 6 min of walking test distance (6 MWTD) was improved in the exercise group [32]. Böhm et al., studied the effect of a 30 min cycling exercise at 60–70% HRmax on solute removal, blood gases, and oxidative stress. The results showed that the level of soluble substances increased with exercise but only a significant increase in phosphate was observed. Oxygen partial pressure (pO2), as well as oxygen saturation, increased with exercise, which was not the same in the control group. Finally, the results showed that the antioxidant capacity decreased due to this type of exercise [7]. Patients with ESRD may experience circulatory overload during hemodialysis. Accordingly, Brown et al., studied the effect of cycling at both 55% and 70% of HRmax on urea balance and clearance (mL/min) in patients with ESRD. The results showed that a higher intensity had no additional effect on urea clearance. Additionally, urea clearance was higher in both exercise groups with 55% and 70% HRmax compared with the non-exercise group [33]. A high level of uremic toxins in patients with ESRD causes uremic toxicity and impairs erythropoietin (EPO) synthesis. This deficiency results in a defective erythropoiesis due to a decrease in red blood cell (RBC) production. A lack of EPO along with iron deficiency are the factors that contribute to anemia [34]. Aerobic exercise also reinforces the antioxidant defense system, which is reduced in dialysis patients due to the excretion of uric acid and antioxidant vitamins. [35]. Vaithilingam et al., examined the effect of exercise and time on dialysis performance in patients with KD. Subjects were divided into the exercise and control group with increased dialysis time (longer dialysis). The results of the study showed that aerobic exercise and increasing the time of dialysis can lead to increased phosphate removal [36]. In another study, Sakhaee et al., investigated the effect of exercise with intensity equal to 70–75% of VO_2max_ on the formation of renal stones. The results of this study suggested that due to the increased crystallization of uric acid and calcium oxalate, moderate-intensity exercise increases the potential for renal stone formation without adequate fluid intake [37]. In another study, Fuhro et al., examined the effect of an exercise session during hemodialysis on the subsets of natural killer (NK) cells. Twenty minutes of moderate-intensity exercise (intensity equal to 6–7 on 10-point Borg RPE scale) had no significant effect on the NK cell count. No changes were observed in the subtypes of NK cells, as well as creatine kinase enzymes and C-reactive protein [11].

The prevalence of poor physical function, which is related to poor health-related quality of life, and an increased risk of adverse consequences as well as hospitalizations and all-cause mortality, is high in CKD patients. Exercise is one of the best interventions for preventing a decline in physical function. Baggetta et al., investigated the effects of a training program on quality of life, body profile, and physical performance in dialysis patients. Subjects were divided into active (home-based, low-intensity program) and control (usual) groups. The physical assessment included 6 MWTD and the five-times sit-to-stand (5 STS) test. After the end of the 6-month training period, the results showed improvement in the 5 STS test (2.7 s decrease) and 6 MWTD (distance increased as much as 39 m) in the active group, but in the control group, which received their usual care, no change was observed [38]. In addition, Bogataj et al., performed a study of intradialytic exercise on a bicycle with functional exercises in this group. The functional exercises consisted of a 16-week, three-sessions-per-week program that covered the whole body in terms of flexibility, strength, endurance, balance, and coordination. The control group in this study only pedaled, while the exercise group performed functional exercise combined with cycling. The exercises started at 15 min and reached 60 min at the end of the training period; they were performed with an intensity equal to 7–8 on the 10-point Borg RPE scale. The results showed that at the end of the 8 weeks, the training group had better upper- and lower-body flexibility, balance, and grip strength than the active control group (cycling only). Furthermore, all items except lower-body flexibility were retained for the functional group [39]. In another study, Parsons et al., studied the effect of exercise on dialysis efficiency and physical function. Exercise was performed for three sessions per week for 5 months. The results showed that dialysis efficiency increased by 11% after the training period. The results also showed that physical function measured by the 6 MWTD improved by 14% [40]. Gomez et al., also examined the long-term effects of aerobic exercise on people with CKD and non-obese dialysis patients. The exercise was performed for three sessions per week for 24 weeks. The measured physical indicators after the training period in the training group increased significantly. Bone alkaline phosphatase (BAP) also increased after 24 weeks. Bone markers including sclerostine and osteocalcin were reduced in this study, indicating an improvement in physical function, as they contrast with muscle and bone mass indices [9].

Cognitive impairment is often noticeable in different cognitive domains: executive function (judgement and planning), attention, language, memory, and visual–spatial learning [41]. An impairment to cognitive function, mainly to executive function, is common in kidney disease (mostly in ESKD patients) and may be aggravated by maintenance dialysis because of the retention of uremic toxins, recurrent cerebral ischemia, and inactivity [42]. Cognitive impairment may cause long-term adverse outcomes, as well as dementia and death [41]. Exercise is a promising approach to protect cognitive function. Tang et al., examined 12 weeks of aerobic exercise for three sessions per week for 20–30 min with an intensity set by the experimenter to achieve a target RPE of 12–15 on psychological dimensions and health-related quality of life. Training started progressively from low to moderate intensity, which included indoor walking, jogging, and cycling. The results showed that all domains of health-related quality of life in the training group significantly improved and anxiety, depression, and psychological stress were significantly reduced after the 12-week training program [6]. In another study, the effect of aerobic exercise on cognitive function in patients with CKD was investigated. The training intervention was a 4-month program with three sessions per week for 30–45 min, each with an intensity equal to 65–75% of the maximum heart rate (HRmax). Cognitive function improved after the training period [10]. Baggetta et al., investigated the effects of a home-based, low-intensity training program on cognitive function in dialysis patients. The physical assessment included the 6 MWTD and the 5 STS test. The results indicated that cognitive function decreased in the control group after 6 months, while it was preserved in the active group [38]. In their study, Dashtidehkordi et al., investigated the effect of 8 weeks of cycling for 30 min with an intensity equal to 14–15 RPE and three sessions per week on health-related behaviors. The results showed that exercise increased physical function and improved stress management [27]. A simple, personalized, home-based, low-intensity walking exercise (exercise group) compared with non-exercise (control group) significantly improved the cognitive function score (*p* = 0.04) and quality of social interaction score (*p* = 0.01) in patients with stage G5 CKD [32].

In another study, Carney et al., examined the effect of exercise (50–60% of VO_2max_) on psychological indicators and depression in dialysis patients. The rate of depression decreased according to the score scale in these patients. The participation of active people increased in the post-workout follow-up period for recreational activities [28].

### 3.2. Resistance Exercise and Kidney Diseases

There is a growing body of evidence demonstrating that resistance training is an important adjunct strategy that provides a multitude of benefits for CKD patients, including a reduced loss of muscle mass and strength, improved inflammatory markers and vascular endothelial function, and reduced oxidative stress [43]. Patients with different kidney diseases, such as AKF (acute kidney failure) and ESRD, and patients with kidney transplantation also develop sleep-related disorders, increased inflammation, redox imbalance, and factors associated with endothelial dysfunction [11,12,24,34,44]. In their study, Corrêa et al., examined the effect of resistance training on sleep quality, redox balance, and inflammatory markers. In this study, individuals performed resistance training for 12 weeks, with three sessions per week and 50 min per session. Exercises were conducted in the upper and lower limbs and RPE was in the range of 6–8 based on the OMNI Resistance Exercise Scale (OMNI-RES). The exercise was performed for three sets of 8–12 repetitions with 2 min rest intervals between sets. The results showed that resistance training improved the asymmetry of dimethyl arginine (ADMA) and thus increased the bioavailability of nitric oxide (NO) and improved the state of redox and sleep quality, which resulted in reduced oxidative stress, inflammation, and ADMA [35]. Watson et al., investigated the inflammatory response (IL-6, IL-15, MCP-1, TNF-α); the myogenic (MyoD, myogenin, myostatin), anabolic (Akt, eEf2), and catabolic events (MuRF-1, MAFbx, 14 kDa, ubiquitin conjugates); and the overall level of oxidative stress, before and after training (8-week progressive resistance exercise program consisting of three sets of 10 to 12 leg extensions at 70% of estimated one-repetition maximum thrice weekly). This type of training was well tolerated by patients with CKD and granted important clinical benefits. It has been shown that CKD patients exhibit a small molecular response to unaccustomed resistance exercise, which would be significantly increased with training that improves muscular strength and size [43].

Muscle wasting is highly prevalent in CKD and is clinically crucial given its strong association with morbidity and mortality in many other chronic conditions. The reason for this muscle loss has not yet been fully investigated but is likely to be multifactorial, including metabolic acidosis, inflammation, insulin resistance, oxidative stress, and physical inactivity [45]. Cheema et al., studied the impact of 6 weeks of progressive resistance training on muscle indicators (hypertrophy, muscle strength). In this study, seven randomized controlled trials investigated the effect of resistance training on muscle strength (*n* = 249), and six studies examined total muscle mass (number = 220). The results showed that muscle strength improved significantly but total muscle mass did not change [46]. Moreover, this study, using the design of a special device, indicated the possibility to perform resistance training in the upper body during dialysis [46]. In another study, Chan et al., examined the possibility of using a device to implement resistance training within a dialysis session in patients with ESRD. Subjects performed unilateral and bilateral exercises with 2.5 to 59 kg loads in three lower-body and two upper-body exercises for three sessions per week for 12 weeks during and before dialysis sessions. Muscle strength, as well as all subscales related to the quality of life, increased significantly in the progressive resistance training group. Additionally, in this study, it was found that 71% of patients adapted to all loads, and no side effects were reported [47,48].

In their study, Abreu et al., examined the effect of resistance training on nuclear factor B (NF-kβ) as well as nuclear factor erythroid 2-related factor 2 (Nrf2). The results showed that physical, mental, and psychological health increased in the resistance training group. The amount of glutathione peroxidase (GPx) and NF-B increased in the exercise group and the NO concentration was maintained, while it decreased in the control group. As inflammation increased in these patients, antioxidant capacity also decreased and led to the production of reactive oxygen species (ROS), which released the inhibitory subunit of NF kappa B alpha (IkBa) from the NF-kβ and transferred it to the nucleus, leading to the exacerbation of inflammation by the expression of pro-inflammatory cytokines such as interleukin-6 and -8, as well as monocyte chemo attractant protein-1 (MCP-1). As a result, resistance training reduces inflammation and fights ROS by increasing Nrf2, which leads to NAD (P) H Quinone Dehydrogenase 1 (NQO1) overexpression [49]. NF-kβ is a common cross-regulation between immunologic and metabolic paths [50,51], which has a major impact on the transcriptional regulation of genes involved in apoptosis, cell survival, and inflammation. It also acts to differentiate monocytes into osteoclasts [50]. NF-kB controls and regulates metabolism at several levels: it modulates insulin and glucagon signaling and AMP-activated protein kinase (AMPK) activity; controls mTOR activity; interacts with metabolic enzymes such as PDH and PFK; and controls glycolysis, triglyceride levels, lipogenesis, mitochondrial function, autophagy, and protein turnover [52]. This factor can be targeted by several stimuli such as hypoxia, DNA damage, over-nutrition, lipid storage disorders, binding oxidized lipids to vascular endothelia, or endoplasmic reticulum (ER) oxidative stress [50,51]. It has been indicated that NF-κB contributes to several metabolic diseases such as obesity, diabetes, and atherosclerosis [51]. In their study, Moutoula et al., confirmed that MiR-223 (a pleiotropic inflammatory regulator of metabolic-related diseases) acts along with NFkβ. MiR-223 induces the differentiation of monocytes into macrophages. It also plays a key role in CKD, where vascular calcifications and atherosclerosis are present [50].

In their study, Bennett et al., investigated the effect of resistance training at home on patients with peritoneal dialysis. Subjects in this study exercised in three stages with a follow-up stage at 12, 24, and 36 weeks, respectively; the exercise consisted of resistance training, including six exercises in the upper and lower body using thick colored elasticbands (the resistance exercises were made progressively harder using different colour-graded elastic bands) and free weights. The exercise was performed with two sets of 15–20 repetitions in each section. The results of the 30 s sit-to-stand and timed up-and-go test (TUG) improved [53].

Health-related quality of life is a major outcome measure increasingly used in patients with CKD. These patients demonstrate relatively poor physical functioning and quality of life when compared to healthy peers [54]. It is recommended for them to perform regular physical activity from the initial stages to boost their physical and psychological condition and reduce mortality and improve quality of life [55]. Cheema et al., studied the impact of progressive resistance training on the quality of life. In this study, six investigations were carried out into the quality of life (number = 223). The results showed that quality of life significantly improved [46].

Patients with CKD usually develop mineral and bone disorders because the rate of bone resorption exceeds the rate of bone formation, resulting in a loss of bone quantity and quality. Systemic alterations generated by the disease include reduced levels of vitamin D and osteocalcin (OC) carboxylation, and elevated serum PTH and fibroblast growth factor 23 (FGF-23), which are the main regulating hormones of bone integrity and mineral homeostasis [56]. Weight-bearing or resistance exercises have been proposed as a potential stimulator of resident osteocytes to yield signaling molecules that regulate bone formation and bone resorption [57,58]. In a recent systematic review of observational and experimental studies, there was a positive association between resistance exercise and bone mineral density at the femoral neck and proximal femur, with improved bone formation and inhibited bone resorption [59]. In their study, Marinho et al., examined the influence of resistance training during dialysis sessions on osteoblast activity. People in the training group practiced resistance training for 8 weeks. The results showed that bone alkaline phosphate, as well as 1,25-Dihydroxy vitamin D, increased after exercise. Moreover, serum levels of sclerostine and bone alkaline phosphate conflicted with exercise, suggesting that resistance training in hemodialysis patients stimulates bone alkaline phosphate and can be used as a factor to prevent bone loss and bone stimulation [60]. The data are summarized in Table 2.

### 3.3. Combined Exercise and Kidney Diseases

Few studies have been conducted on the effects of combined (aerobic and resistance) exercise training on CKD patients. Regular aerobic exercise has favorable effects on improving cardiorespiratory fitness and health and muscular strength, while resistance exercise opposes protein energy wasting, sustains muscle mass, and increases strength [61]. As a result, the best exercise protocol for these patients is still controversial.

In a randomized controlled trial study on hemodialysis patients, the combined training (10 weeks) was more effective than resistance training alone at improving functional performance (6 MWTD) [62]. A recent meta-analysis confirmed the hypothesis that combined aerobic and resistance exercise is beneficial for improving renal function in adult patients with CKD. There was a significant improvement in the eGFR and serum creatinine levels and a decline in the systolic and diastolic blood pressure. However, no significant differences were demonstrated in proteinuria, lipid levels, physical composition, and quality of life. The results support the idea that combined exercise improves renal function [63]. Watson et al. (2018) investigated whether combined exercise (aerobic plus leg extension and leg press exercise) would elicit greater adaptations (improved cardiorespiratory fitness and cardiac function together with increased muscle mass and strength) compared with aerobic training alone (treadmill, rowing, or cycling exercise) in non-dialysis CKD. The addition of resistance exercise to aerobic exercise conferred greater increases in muscle mass and strength than aerobic exercise alone [6].

In several studies, researchers have examined the effects of combined exercises on the performance of hemodialysis patients. In one of these studies, the effects of 24 weeks of exercise on blood pressure, quality of life, dialysis efficiency, and exercise capacity were studied. The subjects in the control group performed stretching exercises in the upper and lower body for 15 min to give the effect of the sham treatment. The exercise was performed in six stages., Subjects performed aerobic exercise for 20-min and resistance exercise for 10-min in the first stage. Aerobic exercise was continued 15-min in the second stage, 10-min in the third and the fourth stage, 15-min in the fifth stage, and 20-min in the last stage. Resistance training time was also adjusted according to the total training time. The results showed that dialysis adequacy increased by 13.2% and systolic and diastolic blood pressure decreased by 8.5 and 6.5 mmHg, respectively, but the quality of life did not change. Possible mechanisms for these effects include increased blood flow, resulting in an increase in the surface area, and the release of toxins and urea into the bloodstream, resulting in its excretion [13]. In another study, Uchiyama et al., examined the effect of 12 weeks of home-based training (aerobic exercise and resistance training, two and three sessions per week, respectively) on quality of life and physical function. Indicators related to individual as well as psychological effects in the exercise group increased compared to the control group, and physical function also increased according to the walking test. The results of this study also showed that the amount of albumin was preserved. The progressive walking test, which indicates aerobic capacity, also improved in this study [64]. In another study, Watson et al., found that combined aerobic and resistance training had a greater impact on CKD than aerobic training alone. In this regard, Cho et al., compared the effect of three methods of resistance, aerobic, and combined training on the quality of sleep and daily activities. The results showed that the metabolic equivalent (MET) significantly increased in the aerobic and combined groups and when the comparison was made between the groups, and the highest increase was observed in the combined training group. The sleep degradation index decreased in the aerobic and resistance exercise groups. Sedentary days were also reduced in all exercise groups after 12 weeks [6,44]. Barcellos et al., investigated the effect of 16 weeks of aerobic and resistance training on the blood glucose, C-reactive protein, blood pressure, eGFR, and lipid profile. The results showed a significant decrease in fasting glucose and C-reactive protein, and an increase in the functional capacity [2]. Hiraki et al., studied the effect of home-based aerobic and resistance exercise (brisk walking for 30 min per day for 12 months and handgrip and knee-extension muscle strength) on pre-dialysis CKD patients. These exercises were feasible and improved arm and leg muscle strength. The handgrip strength (17%) and knee extensors (8.2%), as well as physical activity in the exercise group, improved among the pre-dialysis patients. Kidney function (GFR) did not differ between groups after the training period [65]. This lack of change in the GFR was confirmed in a study by Hamada et al., who applied aerobic and resistance exercise. In this study, an increase in the MET was also reported in the exercise group [4]. In another study, Van Bergen et al., included 20 children with a history of ESKD. Seventy percent of the children could not complete the exercise protocol. Participants trained for 12 weeks and practiced two sessions per week. The training protocol included 50 min of aerobic training with an intensity of 55–90% VO_2_peak. Resistance training and active games were also part of the training protocol. In the final four weeks, 10 min of interval training was added to the protocol. The results of the study showed an improvement in VO_2_peak as well as muscle strength. The researchers suggested that this training protocol is difficult and not applicable in children with ESKD [66]. In another study, Thompson et al. examined the effect of chronic aerobic training combined with isometric contraction (24 training sessions) on blood pressure in 160 CKD patients. The results showed a reduction in blood pressure [67].

In one study, 151 non-dialysis CKD patients conducted self-administered exercise training (150 min per week for 4 months), consisting of 60 min of endurance training in combination with 90 min of either strength or balance training (strength versus balance group). Overall endurance (6 MWTD, stair climbing); muscular endurance (30 STS, heel rises and toe lifts, handgrip and isometric quadriceps strength); balance (functional reach and Berg’s balance scale); and fine motor skills (Moberg’s picking-up test) were measured pre- and post-training. Two different exercise training programs (endurance in combination with either strength or balance exercises), improved muscular strength and endurance, balance, and motor skills after a 4-month training period in CKD patients, regardless of age and comorbidity [68].

### 3.4. Blood Flow Restriction (BFR) Exercise and Kidney Diseases

In the last decade, researchers have gone a step further and examined the impact of new training methods, such as exercise with blood flow restriction, on patients with ESKD. In one of these studies, Clarkson et al., included 10 dialysis patients in one of the following three conditions: two consecutive 10 min cycling sessions in two dialysis sessions, two separate cycling sessions with BFR on non-dialysis days, and two cycling sessions with BFR on dialysis days. Heart rate, blood pressure, and clearance time were also measured. They reported that systolic, diastolic, and mean arterial blood pressure decreased by 12, 5, and 11 mmHg, respectively, over a 60 min recovery period. This reduction returned to normal levels after the recovery period, and the BFR did not affect the volume of clearance time (kt/v). The researchers reported that exercise with BFR was comparable to the standard aerobic exercise in terms of hemodynamic safety and tolerability. It is noteworthy that the researchers reported that one of the patients in the exercise group suffered from syncope with BFR and his condition improved after stopping the filtration. The researcher also reported that the mentioned cases were overloaded with fluids and ultrafiltration was prescribed for them. In this study, it was also suggested that the ratio should be considered as 1.4 to ensure dialysis adequacy [69]. In this regard, Cardoso et al. examined the effect of intradialytic exercise with BFR and compared it with traditional exercise. Three equal groups (aerobic exercise, control, and BFR) were examined. The 6 MWTD in the BFR group was reduced compared to the traditional method and the control group [70]. The data are shown in Table 3 and Table 4.

### 3.5. High-Intensity Interval Training and Kidney Diseases

High-intensity interval training (HIIT) (≈85% VO_2max_) has been the focus of several studies [21,71], which hypothesize that HIIT may induce significant benefits compared to traditional moderate-intensity continuous exercise (MICE) (≈50% VO_2max_). The improved cardiovascular [72] and metabolic [73] consequences induced by HIIT are the reason for this hypothesis. In addition, HIIT is also safe for many high-risk populations [72], even at an advanced age [74], including patients with CKD [18], which makes HIIT an attractive therapeutic strategy in CKD patients. However, there is a lack of human studies, and most of the research studies have been conducted on animal models; as animal research may be a poor predictor of human experience, these papers were not included in this study.

Nilsson et al., studied the effects of 22 weeks of HIIT compared with moderate-intensity continuous training (MICT) on dialysis patients. Exercises were performed for two sessions per week with an intensity of 50–60% HRmax for 45 min in the MICT group. In the HIIT group, individuals performed three sessions with an intensity of 85–95% HRmax along with 4 min of active rest with an intensity of 60–70% HRmax. The last exercise interval was followed by a 10 min cool-down. The results showed that 55% of the participants completed the exercises. Two out of three individuals increased their peak oxygen consumption by 46% and 53%, respectively. The peak oxygen consumption in three individuals in the MICT group increased (by 6%, 18%, and 36%, respectively). One of the limitations of this study was the small sample size [71]. Beetham et al., conducted a similar study on these patients. Individuals in the HIIT group performed 4 × 4 min intervals with 80–95% HRmax, and in the MICT group, individuals performed 40 min with 65% HRmax. PGC1α was measured as an indicator of mitochondrial biogenesis, MuRF1 as an indicator of muscle catabolism, and Phospho-p70 S6 Kinase (Thr389) as an indicator of muscle protein synthesis. The results showed that HIIT is feasible and safe for CKD patients and that both types of exercise lead to an increase in skeletal muscle protein synthesis capacity as well as exercise capacity [21]. The data are shown in Table 5.

### 3.6. Electrical Muscle Stimulation and Kidney Diseases

Electrical muscle stimulation (EMS) is used as a kind of therapy to reduce muscle mass loss via its effect on muscle activity as well as improving insulin resistance. Moreover, electrotherapy has the potential to enhance kidney function due to circulatory effects as well as lowering the sympathetic tone [75].

According to the literature, hemodialysis patients show poor physical conditioning and low exercise tolerance [76]. They may also suffer from respiratory dysfunctions. As a result, Roxo et al., evaluated the effects of neuromuscular electrical stimulation on the pulmonary function and functional capacity of patients with CKD on hemodialysis. The subjects were randomized into control and treatment groups. The treatment group underwent bilateral femoral quadriceps muscle EMS for 30 min during hemodialysis, three times per week for two months. Electrical neuromuscular stimulation had a positive impact on pulmonary function (maximum respiratory pressures, expiratory pressures) and functional capacity (muscular strength in maximum one-repetition test and distance covered in the 6 MWTD increased), leading to better physical performance in hemodialysis patients [76].

The effect of 1 h intradialytic high-tone electrical muscle stimulation (HTEMS) was evaluated on stage-three acute kidney injury patients, all of whom required daily hemodialysis. HTEMS was well tolerated and improved clinical outcomes. The treatment group presented a significantly shorter duration of oliguria, a faster decline of serum creatinine and urea levels, less need for dialysis treatment, and a shorter period of hospitalization compared with the control group. The decline in urea was more marked than that in serum creatinine, resulting in a significant lowering of the urea/creatinine ratio. The catabolism of muscle proteins was reduced, which led to a lower release of amino acids into the circulation. The improved protein catabolism is probably the reason for the shortening of the clinical course of acute renal failure [75].

Suzuki et al., studied the effect of EMS on the isometric strength of knee extensors as well as the cross-sectional area of the quadriceps muscles. The intervention was performed during the first 2 h of dialysis sessions for eight weeks and three times per week. The excitation frequency was 20 Hz, and the pulse width was 250 ms. Each stimulation cycle consisted of 5 s followed by a 2 s pause period of 20 min in the form of a monophasic, exponential climbing pulse. The results showed a significant increase in the knee extensor strength and the cross-sectional area of the quadriceps muscles. Timed up-and-go test (TUG) performance was also improved [77]. In a similar study, McGregor et al., examined the effect of low-frequency EMS (LF-EMS) on dialysis and cycling exercise in these patients. Subjects were divided into three groups of low-frequency EMS, cycling, and control. Individuals in the training group performed exercises for one hour, three times per week with an intensity of 40–60% maximal oxygen uptake and LF-EMS at the maximum tolerable intensity. At the end of the 10 weeks of training, cardiorespiratory fitness (VO_2max_) increased by 2 and 3 mL/kg/min in the EMS and cycling group, respectively. The results also showed that leg strength after training was higher in the EMS group than the cycling group (95 vs. 65.1, respectively). Accordingly, the use of LF-EMS in these patients is a practical alternative for those unable or reluctant to cycle during dialysis [78]. The data are given in Table 6.

### 3.7. Exercise and Rehabiliation in Patients with Neurogenic Bladder, Polycystic Kidney Disease, and Glomerulonephritis

In recent years, the concept of renal rehabilitation has become well-known. WHO defined rehabilitation as “a set of interventions needed to regain or improve conditions that may bring about disabilities and social disadvantages”. Accordingly, renal rehabilitation was defined as “a long-term coordinated program (exercise, nutrition, etc.) to improve physical/mental effects based on kidney disease and dialysis therapy, improve longevity and psychosocial and occupational circumstances” [80]. Despite the reported positive effects, some researchers did not confirm these results; for example, a study by Cornacoff et al., did not show a significant difference in the rate of hospitalization due to disease in patients conducting exercise intervention (rehabilitation program) and patients in the control group [81]. However, in another study, Andrade et al., examined a course of physiotherapy on a neurogenic bladder, also known as neurogenic lower urinary tract dysfunction, in which the nerves that carry messages back and forth between the bladder and the spinal cord and brain do not work properly.

It is believed that patients with neurogenic bladder have a significantly higher risk of developing CKD compared with the general population [82]. A total of 210 patients with a history of neurogenic bladder (HTLV-1) participated in this study. The movement therapy program included the electrical stimulation of the pelvic floor muscles. The results showed that physiotherapy was effective in reducing symptoms, increasing perineal muscle strength, and improving urodynamic parameters and quality of life [83].

Polycystic kidney disease, the most common heritable renal disease, is a disorder characterized by the development and growth of cysts in the kidneys and other organs [84]. In their study, Martinez et al., studied the effect of exercise on a pedometer bicycle on patients with polycystic KD with normal blood pressure. Patients performed 12 weeks of aerobic exercise on a bicycle. The results showed that due to having a high-quality dialysis session, patients could participate in exercise immediately after the treatment process. Systolic blood pressure was equal in response to exercise as well as in the recovery period. Diastolic blood pressure decreased in response to exercise, but this decrease was lower in the exercise group than in the control group. Patients with polycystic KD had high systolic blood pressure in response to exercise. Moreover, the left ventricular mass in these patients was higher than in the control group. The findings suggested that the increased response of systolic blood pressure to exercise was due to impaired vascular vasodilation in these patients [85]. In another study, Reinecke et al., examined the effect of exercise on hemodialysis efficiency. The GFR was less than 60 mL/min per 1.73 m^2^. The results showed that peak oxygen uptake and anaerobic threshold in patients with polycystic KD was lower than their healthy peers [86]. In their study, Yamagata et al., developed guidelines for providing exercise to patients with KD. In patients with chronic glomerulonephritis with moderate proteinuria (0.8–1.5), exercise led to increased proteinuria, but it returned to baseline 2 h after exercise. The results also showed that after the Bruce stress exercise test, proteinuria increased by 69.5%, but 2 h after the end of the exercise, the level of proteinuria returned to the baseline. Renal function increased by 7.1% after exercise. The results displayed that low levels of hemoglobin, decreased renal function, and proteinuria were higher in patients with low levels of physical activity. VO_2_peak was higher in active patients than sedentary ones and was inversely related to the duration of the disease. The main point is that there was no evidence that exercise therapy worsened these patients’ condition. As it may enhance exercise tolerance, its applicability should be assessed individually according to age and physical function [80]. Glomerulonephritis is inflammation of the tiny filters in the kidneys (glomeruli). Glomeruli remove excess fluid, electrolytes, and waste from the bloodstream and pass them into the urine. Glomerulonephritis can come on suddenly (acute) or gradually (chronic). There is no clear evidence that the prognosis of glomerulonephritis is exacerbated by exercise or that the prognosis is improved by rest and exercise restriction. Therefore, exercise restriction should not be applied to patients with glomerulonephritis [80]. The data are summarized in Table 7.

**Figure 2:**
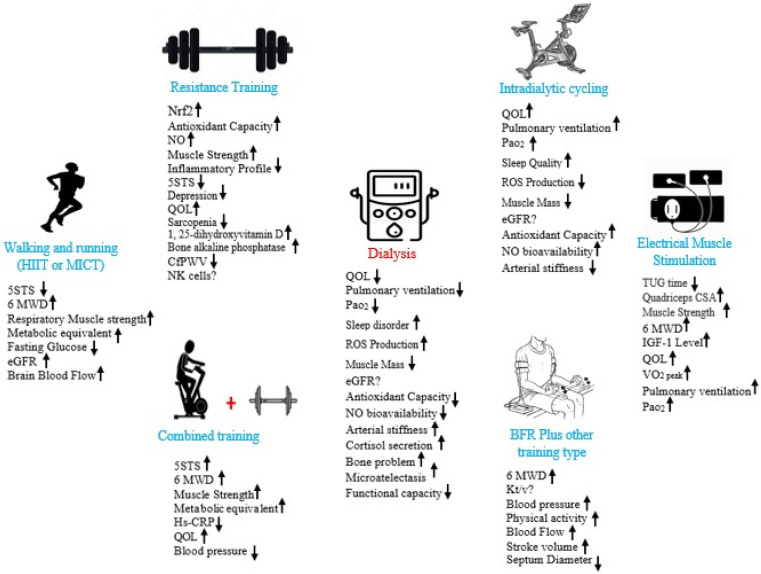
Schematic view relevant to the impact of different types of exercise on kidney diseases.

Possible mechanisms for the effect of different kinds of exercise on kidney function are given in Figure 2.

## 4. Discussion

This review mostly involved studies on patients with ESRD; however, other approaches [2,4,5,6,12,21,65] were carried out in different stages of kidney disease. There is conflicting evidence on the effect of exercise on the biochemical factors related to kidney function and the exact mechanism by which exercise may be beneficial in CKD patients. That being said, the well-known anti-inflammatory response of exercise could be its most crucial effect in these patients [87], since kidney inflammation is prevalent in renal injury [88], which ultimately causes fibrosis and the everlasting loss of function [89]. The mechanism for the strong anti-inflammatory effect of exercise is visceral fat reduction, which decreases the secretion of pro-inflammatory adipokines [87] and increases levels of anti-inflammatory cytokines (interleukin-6 (IL-6), from contracting skeletal muscle) [87]. An increase in IL-6 levels slows the rise in tumor necrosis factor-alpha (TNF-a) levels after exposure to an inflammatory stimulus [90]. The exercise-induced rise in IL-6 and the simultaneous reduction in TNF-a levels proposes the potential therapeutic effect of exercise in controlling low-grade inflammation, which can deteriorate CKD [89]. In fact, inflammation, insulin resistance, oxidative stress, and an overactive renin-angiotensin system preserve each other while negatively affecting endothelial and cardiovascular function and health [91]. Exercise improves vascular insulin signaling, raises bioavailable NO, and decreases ROS [1]. At the molecular level, NO production increases due to exercise through a sequence of increasing both NO precursor molecule L-arginine availability and the activity of endothelial NO synthase (eNOS) and decreasing NO degradation through decreasing ROS [2]. Exercise promotes the endothelial response to insulin by decreasing angiotensin II and other inflammatory molecules such as TNF-α [1]. A wide range of studies support the positive role of aerobic, resistance, and combined training, with BFR, HIIT, EMS, and functional training in improving renal function as well as developing the quality of life in patients with KD. Many of the studies examined the effect of different types of exercise on the quality of life and functional capacity of patients with different stages of KD. Most of these studies used TUG, 5 STS, 30s STS, and 6 MWD as indicators of functional capacity, and most of them confirmed the positive effect of exercise on functional capacity [4,6,27,53,64,65,71,77]. In all of these studies, the STS, as well as the TUG test time, were reduced, and the walking distance was increased within six minutes. Sleep quality was also assessed using the sleep fragmentation index and ADMA. The results showed improvements to the sleep pattern in the exercise group in both studies compared with the control group [35,44]. Exercising with adequate fluid intake reduces the risk of waste product accumulation and decreases the risk of kidney stone formation. Exercise improves peak oxygen consumption in patients and also decreases blood pressure and improves health-related indicators such as lipid profiles and inflammatory indicators as well as psychological indices such as depression and anxiety [92]. Studies on blood hemodynamics [2,10,12,13,23,25,77] have used different indices such as flow-mediated vasodilation (FMV), CFPWV, and arterial stiffness (AS). In some studies, refs. [10,12,44] hemodynamic improvement was demonstrated, but in other studies, no change was observed in FMV, CFPWV, and AS [23,25,78]. Exercise in different stages of KD leads to a temporary change in fecal matter (proteinuria), which returns to a normal level during the recovery period. Exercise during a dialysis session also results in a temporary decrease in blood flow that returns to a normal level during the recovery period. Oxidative stress, pro-inflammation, and malnutrition occurring in CKD lead to the accumulation of metabolic waste products and alterations in homeostasis, which affect many target organs, including the cardiovascular system, and reduce physical capacity, which alters osteoporosis and the loss of muscle mass and ultimately leads to death [93]. Sarcopenia is present from the early stages of CKD and its prevalence rises in the more advanced stages, which is related to higher mortality and disability rates and an increased risk of falls, fractures, and hospitalization [94]. If exercise is designed according to the age range and is individualized for each patient, it will lead to significant improvements. Exercise should be limited in children with chronic renal disease. The only present study in this case showed that the exercise load is not tolerable for 70% of children [66]. Therefore, exercise prescription in this group should be considered with caution. There is not sufficient evidence to support the idea of physical activity prohibition in patients with glomerulonephritis, nephrotic syndrome, and kidney transplantation. Given the wide range of studies on the positive role of exercise (aerobic, resistance, and combination) in improving the condition of patients with acute and chronic renal disease, some studies suggest that strenuous exercise, especially when combined with low water intake and in hot weather, can in some cases lead to acute kidney damage and failure (in healthy people) [95]. In intense competitions, the severe rupture of muscle tissue (rhabdomyolysis) releases toxic intracellular components into the systemic circulation, which leads to an overload of renal tubules, heat stress, and intense sympathetic nerve activity due to myoglobin. The potential benefit of aerobic exercise to reduce blood pressure and improve the lipid profile in these patients has been indicated, and it is suggested that the diet should be adjusted along with aerobic exercise. Considering the support of studies on the effect of resistance training on hypertrophy and the strength of different muscle fibers in CKD patients [6,35,39,44,46,48,51], resistance training is suggested in patients with kidney disease. Studies have also shown that exercise (aerobic, resistance, etc.) improves glomerular filtration and reduces the risk factors for cardiovascular disease. Several studies have examined redox balance, inflammation index and total antioxidant capacity [7,11,12,35,49]. The results of these studies in the field of antioxidant capacity are ambiguous, because this factor was reduced in one study [7] but improved in another study [49]. Inflammation markers improved in all of the mentioned studies except the study of Fuhro et al., [11]. The redox balance improved in all of these studies [7,11,12,35,49]. It seems that the observed improvement in redox status, as well as the reduction in inflammation, is achieved due to the reduction in uremic toxins through increased dialysis efficiency and the excretion of soluble substances. Increased dialysis efficiency was studied by measuring the excretion of soluble substances and the results of the studies proved the increase in excretion of these soluble substances after exercise or an increase in the dialysis time [13,26,33]. On the other hand, some studies indicated that the levels of substances such as albumin and phosphorus do not increase or were maintained, which may be related to the intensity and type of exercise used in the study. In the study of Böhm [7] et al., no increase in dialysis efficiency was observed. The current exercise guidelines also suggest that people perform stretching exercises for 10 min each day along with other exercises to develop flexibility. Exercise increases the total antioxidant capacity of the system by affecting transcription factors [96]. It also helps improve blood pressure by increasing the bioavailability of NO and improving the vasoactive balance, as well as reducing the sympathetic activity of vascular smooth muscle [97]. Exercise affects the rate of protein synthesis in muscle by phosphorylating P70^S6k^, thereby reducing the rate of muscle loss and in turn performance in the individual. Finally, according to the results of various studies, it seems that to achieve the benefits of exercise, training should be performed over a period of at least six months. It should also be noted that due to the condition of the parathyroid glands as well as hypogonadism in these patients, conducting any contact sports or exercises with a high workload (excessive repetition) is not recommended [98], because it leads to increased kidney damage in these patients.

## 5. Conclusions

To sum up, the majority of previous studies showed that: (a) there are beneficial effects of various types of exercise on different kidney diseases, whether during or outside of dialysis session; (b) chronic aerobic, resistance, or combined training, if accompanied by safety considerations, seems to have many benefits for patients with KD, such as improved glomerular filtration, reduced cardiovascular risk factors, increased maximal oxygen uptake, improved muscle protein synthesis, increased or maintained strength, improved body composition, enhanced quality of life, and other health-related factors; (c) the type and stage of kidney disease and the patient’s condition should be considered prior to prescribing any exercise program; (d) on the other hand, in CKD patients, exercise prescription should be based on exercise training principles including individualization, specificity, adaptation, recovery, reversibility, and overload; and (e) exercise load is a combination of the frequency, intensity, and duration of the exercise performed and must be considered for any prescription.

## Figures and Tables

**Table 1:** Aerobic exercise and kidney diseases.

Author, Year	Country	Sample Size		Age (Years) Mean ± SD		Duration of Dialysis (Months) Mean ± SD		Time	CKD Stage	Duration	Frequency	Intervention	Outcomes
		I	C	I	C	I	C						
Dashtidehkordi et al. (2019) [27]	Iran	24	22	51.22	55.64	5.48	4.48	Intradialytic	HD	8 weeks	3 times/week	Two half hours with 5 min intervals using a stationary bicycle; the intensity was self-selected	Stationary bicycle during hemodialysis could enhance health-promoting behaviors
Böhm et al. (2017) [7]	Brazil	11	12	52 ± 5	53 ± 3	20 (8–64)	19 (10–45)	Intradialytic	HD	1 session	--	30 min aerobic exercise with intensity between 60–70% of HRmax	Increased phosphorus serum concentration and decreased total antioxidant capacity, increased oxygen partial pressure and saturation, no change in acid base
e Silva et al. (2019) [24]	Brazil	15	15	58 ± 15.0	50 ± 17.2	26.0 ± 14.58	21.0 ± 27.1	Intradialytic	HD	3 months	3 times/week	30 min without interruption, between 65% and 75% of the HRmax	Significant improvement in flow-mediated vasodilation, reduction in left-ventricular hypertrophy and serum aldosterone
Gomes et al. (2017) [9]	Brazil	24	15	55.5 ± 8.3	55.5 ± 8.3	-	-	Home-based	Obese P-HD	24 weeks	3 times/week	30 min 3 times per week with increments of 10 min in duration every 4 weeks until week 8	Aerobic training did not promote relevant changes in the bone metabolism markers
Belik et al. (2018) [10]	Brazil	7	8	50.3 ± 17.24	57.8 ± 15.01	26.0 ± 14.58	21.1 ± 27.10	Intradialytic	HD	16 weeks	3 times/week	30 min with training range of 65–75% HRmax	Significant improvement of cognitive impairment and basilar maximum blood flow velocity in trained patients

AE—aerobic exercise; P-HD—pre-HD; HD—hemodialysis.

**Table 2:** Resistance exercise and kidney diseases.

Author, Year	Country	Sample Size		Age (Years) Mean ± SD		Duration of Dialysis (Months) Mean ± SD		Time	CKD Stage	Duration	Frequency	Intervention	Outcomes
		I	C	I	C	I	C						
Corrêa et al. (2020) [35]	Brazil	30	25	66.0 ± 4.0	65.7 ± 3.8	60.7 ± 8.0	59.8 ± 7.7	Intradialytic	HD	3 months	3 times/week	Consisted of 11 exercises with 2 weeks of familiarization; 3 sets of 8–12 repetitions with 2 min of rest between sets	Decreased ferritin, sleep efficiency improvement
Lopes et al. (2019) [47]	Brazil	30	20	(MLG) 56.2 ± 12.5 and 48.1 ± 10.8 (HLG)	56.9 ± 12.4	(MLG) 72.1 ± 50.3 and 45.7 ± 39.3 (HLG)	53.2 ± 44.1	Intradialytic	HD	12 weeks	3 times/week	Each session involved 5 exercises; exercise was performed until volitional fatigue; the duration of the sessions varied between 20 and 40 min	Increased lean leg mass; improvements in pain and physical function; prevalence of sarcopenia was reduced by 14.3% and 25%; and no change in cytokines was observed
Abreu et al. (2017) [49]	Brazil	25	19	45.07 ± 15.2	42.5 ± 13.5	71.2 ± 45.5	70.1 ± 49.9	Intradialytic	HD	12 weeks	3 times/week	Elastic bands ranged from 1.6 to 10.0 kg, and the load used in exercises performed with ankle cuffs ranged from 1.0 to 12.0 kg; intensity set at 60% of 1RM, since CKD patients are mostly debilitated	Resistance exercises are able to induce Nrf2 activation in CKD patients on HD
Bennett et al. (2016) [53]	Australia	171	-	68.1 (12.6)	-	44 (26.0–85.5)	-	Intradialytic	HD	12 to 36 weeks	2 times/week	Two sets of 15–20 repetitions for each exercise; the resistance exercises were made progressively harder using different color-graded elastic bands; training consisted of six lower- and upper-body resistance exercises	Exercise training led to significant improvements in physical function as measured by STS and TUG
Chan et al. (2016) [48]	Australia	22	-	In total 71.3 6 11.0		In total 24 (15–56)		Intradialytic	HD	26 weeks	2 times/week	2 upper-body and 3 lower-body exercises, unilaterally and bilaterally, both before and during dialysis, with loads of 2.5 to 59 kg	Lower body strength and health-related quality of life (HRQoL) subscales significantly increased and a trend toward reduced depression was noted

RT—resistance training; HD—hemodialysis.

**Table 3:** Combined exercise and kidney diseases.

Author, Year	Country	Sample Size		Age (Years) Mean ± SD		Durationof Dialysis (Months) Mean ± SD		Time	CKD Stage	Type	Duration	Frequency	Intervention	Outcomes
		I	C	I	C	I	C							
Huang et al. (2020) [13]	China	*n* = 16	*n* = 16	43.81 ± 10.25	37.63 ± 10.31	26 (29.75)	43 (89)	Intradialytic	HD	CE	24-week	3 times/week	5 min warm-up, cool-down, and 30 min cycling at a RPE of 12–14	Systolic and diastolic blood pressure significantly decreased by 8.5 and 6.5 mmHg, respectively
Van Bergen et al., 2009 [66]	Netherlands	20	_	Ranged between 8 and 18 yrs, mean 17.6		A history of ESRD treated with either HD or PD for more than 3 months			HD	AE, RE, and game	12 weeks	2 times/week	50 min sessions including 5 min warm-up period; each session involved aerobic training (with an intensity ranging from 55 to 90% HRmax), resistance exercises with no heavy load, and active games	Exercise capacity and muscle strength were higher after the exercise program in patients who completed training
Uchiyama et al. (2019) [64]	Japan	24	23	64.9 ± 9.2	63.2 ± 9.5	Unclear	Unclear	Home-based	PD	RE and AE	24-week	3 times/week (AE), 2 times/week (RE)	AE at 40–60% of the HRmax as determined in the baseline ISWT, with an RPE of 11–13; the program started at 20 min/session and progressed to 30 min/session; RT was prescribed at 70% (1RM) to train a variety of upper- and lower-body muscle groups	Physical functioning, emotional functioning, and role/social component summary significantly improved in the exercise group; serum albumin was maintained in the exercise group
Watson et al. (2018) [6]	UK	21 AE	20 RE	63 (58–71)	63 (51–69)	Unclear	Unclear	Unclear	CKD patients (stages 3b–5)	CE and AE	12-week	3 times/week	30 min of moderate intensity exercise at 70–80% HRmax in CE, and only 20 min of AE were performed; resistance exercise consisted of leg extension; training load (in kg) was set at 70% 1RM, and patients performed 3 sets of 12–15 repetitions and 2–3 min rest interval	Combination of resistance and aerobic exercise confers more increases in muscle mass and strength than aerobic exercise alone
Barcellos et al. (2018) [2]	Brazil	76	74	65.0 (1.2)	65.1 (1.3)	Unclear	Unclear	Gym	CKD patients (stages 2–4) with hypertension	RE and AE	16-week	3 times/week (AE)	10 min of initial warm-up, 60 min physical exercise sessions	Significant decreases in hs-CRP and fasting blood sugar, and increase in functional capacity in exercise group
Cho et al. (2018) [44]	South Korea	33	13	51 ± 22	55 ± 64	54.8 ± 96.4 (AE), 47.6 ± 79.2 (RE) and 87.8 ± 70.5 (CE)	61.4 ± 36.5	Intradialytic	HD	AE, RE, and CE	12-week	3 times/week	AE at 60–70% of an individual’s maximal capacity, RE program consisted of seven exercises for 3 sets of 10–15 repetitions (RPE 13–15), CE group performed both the AE and RE	Improvement in daily physical activity and sleep quality, increase in metabolic equivalent but not to RE, and decrease in sedentary bouts
Thompson1 et al. (2019) [67]	Canada	80	80	Older than 18 yrs	More than 18 yrs	Unclear	Unclear	Home-based exercise	eGFR (15–44 mL/min per 1.73 m^2^) with hypertension (SBP > 130 mmHg)	AE and RE	24-week	3 times/week	Moderate-intensity aerobic exercise (50–60% heart rate reserve) supplemented with isometric resistance exercise in two phases: (1) supervised, facility-based, weekly, and home-based sessions (8 weeks); (2) home-based sessions (16 weeks)	A decrease in blood pressure in response to aerobic exercise and isometric contraction was observed and showed that aerobic exercise followed by isometric resistance training is feasible for patients with CKD

CE—combined exercise; RE—resistance exercise; AE—aerobic exercise; PD—peritoneal dialysis; HD—hemodialysis; eGFR—estimated glomerular filtration rate; SBP—systolic blood pressure.

**Table 4:** Blood flow restriction exercise and kidney diseases.

Author, Year	Country	Sample Size		Age (Years) Mean ± SD		Durationof Dialysis (Months) Mean ± SD		Time	CKD Stage	Type	Duration	Frequency	Intervention	Outcomes
		I	C	I	C	I	C							
Clarkson et al. (2020) [69]	Australia	*n* = 10	-	Unclear	Unclear	Unclear	Unclear	Intradialytic and off hemodialysis	ESKD (stage V); eGFR < 15 mL·min^−1^·1.73 m^−2^; HD > 12 wk	BFR + AE and AE	2 sessions	-	5-min cycling warmup and cool-down at a self-selected cadence; two 10 min bouts of cycling separated by a 20 min rest period; workload for each 10 min bout was between 10 W and 30 W	BFR did not affect ultrafiltration; BFR is comparable to standard aerobic exercise
Cardoso et al. (2020) [70]	Brazil	BFREG (*n* = 19), CEG (*n* = 20)	20	BFREG, 49.4 ± 15.9–59.8 ± 16.1 CEG,	48.2 ± 13.6	54 (17.8–87) BFR-CEG 25 (12–69)	33 (9–52.5)	Intradialytic	HD	BFR + AE and AE	12 Weeks	3 times/week	CEG 20 min training session, intensity increase from 60% to 76% HRmax BFR caused 50% reduction in arterial blood flow during 12 weeks of training	Increased walking distance in BFR+AE group in comparison with two other groups

BFR—blood flow restriction; AE—aerobic exercise; eGFR—estimated glomerular filtration rate; ESKD—end-stage kidney disease; HD—hemodialysis.

**Table 5:** Comparison of high-intensity interval training and moderate-intensity continuous training on kidney diseases.

Author, Year	Country	Sample Size		Age (Years) Mean ± SD		Durationof Dialysis (Months) Mean ± SD		Time	CKD Stage	Type	Duration	Frequency	Intervention	Outcomes
		I	C	I	C	I	C							
Nilsson et al. (2019) [71]	Norway	14	6	59.5 (55–67)	67 (51–69)	Unclear	Unclear	Intradialytic	HD	HIIT and MICT	22 weeks	2 times/week	HIIT or MICT cycling two times per week for a total of 32 sessions over 16–22 weeks; the exercise intervention lasted 45 min	Increased VO_2_peak
Beetham et al., (2019) [21]	Australia	14	-	Ranged between 18 and 75 yrs		Unclear	Unclear	Unclear	Sage 3–4 CKD patients	HIIT and MICT	12 weeks	3 times/week	HIIT performed in 4 × 4 min intervals with 80–95% HRmax and MICT performed as 40 min run with 65% HRmax	There was no significant difference between HIIT and MICT in muscle protein synthesis and time efficieny of training

HIIT—high-intensity interval training; MICT—moderate-intensity continuous training; HD—hemodialysis.

**Table 6:** Electrical muscle stimulation and kidney diseases.

Author, Year	Country	Sample Size		Age (Years) Mean ± SD		Durationof Dialysis (Months) Mean ± SD		Time	CKD Stage	Type	Duration	Frequency	Intervention	Outcomes
		I	C	I	C	I	C							
Suzuki et al. (2018) [77]	Japan	13	13	662.6 ± 12.8	651.6± 8.1	281.6 ± 24.2	304.6 ± 23.6	Intradialytic	HD	EMS	8 weeks	3 times/week	EMS protocol was performed 3 times a week for 8 weeks in lower extremity; waveform simulation produced co-contractions in the lower extremity muscle groups at a frequency of 20 Hz with a pulse width of 250.l s; each duty cycle included a 5 s stimulation period with a 2 s pause for a period of 20 min	EMS could be an effective exercise training tool for HD patients with either muscle wasting, weakness, or sarcopenia
McGregor et al. (2018) [78]	UK	33	18	C- 52.1 [44.2; 59.9] LF-EMS 51.5 [42.3; 60.6]	54.3 [46.0; 62.5]	Unclear	Unclear	Intradialytic	HD	EMS	10 weeks	3 times/week	Two weeks of familiarization allowed participants to become accustomed to the sensation of LF-EMS and progress to at least 30 min of stimulation; cycling was performed for up to one hour per session (minimum of 50 min), initially at a workload (Watts) equivalent to that achieved at 40–60% VO_2_ reserve during CPET; a five min warmup and cool-down	Cardio-respiratory reserve (VO_2_peak) and leg strength were improved; arterial structure and function were unaffected.
Brüggemann et al. (2017) [79]	Brazil	51 in total		Unclear	Unclear	Unclear	Unclear	Intradialytic	HD	NMS	12 sessions	3 times/week	Neuromuscular electrical stimulation with 50 Hz and 24 medium intensity of 72.90 mA, and LG used 5 Hz and medium intensity of 13.85 mA	Increased 6 MWTD in both groups and improved physical capacity

NMS—neuromuscular stimulation and EMS—electrical muscle stimulation; HD—hemodialysis.

**Table 7:** Exercise and rehabilitation in patients with neurogenic bladder, polycystic kidney disease, and glomerulonephritis.

Author, Year	Country	Sample Size		Age (Years) Mean ± SD		CKD Stage	Type	Duration	Frequency	Intervention	Outcomes
		I	C	I	C						
Andrade et al. (2016) [83]	Brazil	21	-	54 ± 12	-	HTLV-1-infected individuals with NB	EMS	10–40 sessions	2 times/week	Low-frequency biphasic current of 12 Hz, 0.2 milliseconds for 30 min; medium-frequency, (50 Hz, 250 μs), with an intermittent 3 s stimulus followed by 1 s of rest for 30 min	Reduction in the overactive bladder symptom score from 10 ± 4 to 6 ± 3; increase in the perineal muscle strength and improvement in symptoms of urinary urgency, frequency, incontinence, and nocturia
Reinecke et al. (2014) [86]	Brazil	26	30 healthy subjects	Ranged between 19 and 39 yrs		ADPKD	AE	1 session	-	20 min single bout of cycle ergometer exercise with 60% of VO_2_peak was performed	Young, normotensive patients with polycystic KD and preserved kidney function had inadequate responses of nitric oxide and ADMA levels to acute exercise compared to healthy subjects; additionally, low aerobic capacity was observed in polycystic KD patients
Yamagata et al. (2019) [80]	Japan	Unclear	Unclear	Ranged between 10 and 69 yrs		Glomerulonephritis, nephrotic syndrome, non-dialysis-dependent CKD	Most include AE	Different	Different	Various types of exercise included Bruce stress treadmill test and bicycle	Temporarily increased proteinuria; results showed a 7.1% increase in renal function after exercise. additionally, a higher VO_2_peak was observed in active patients

HTLV-1—Human T-lymphotropic virus; NB—neurogenic bladder; ADPKD—autosomal dominant polycystic kidney disease.
